# Phosphorylated TDP-43 and tau deposition around the tip of deep brain stimulation leads

**DOI:** 10.1016/j.ensci.2025.100568

**Published:** 2025-05-13

**Authors:** Goichi Beck, Yuki Yonenobu, Kenichiro Maeda, Satoru Oshino, Haruhiko Kishima, Masanori P. Takahashi, Hideki Mochizuki, Shigeo Murayama

**Affiliations:** a Department of Neurology, Osaka University Graduate School of Medicine, Suita, Japan; b Department of Neurosurgery, Osaka University Graduate School of Medicine, Suita, Japan; c Department of Neurology and Neuropathology (Brain Bank for Aging Research), Tokyo Metropolitan Geriatric Hospital and Institute of Gerontology, Tokyo, Japan; d Brain Bank for Neurodevelopmental, Neurological and Psychiatric Disorders, Molecular Research Center for Children's Mental Development, United Graduate School of Child Development, Osaka University, Suita, Japan

**Keywords:** Phosphorylated TDP-43, Deep brain stimulation, Vim nucleus, Subthalamic nucleus, Phosphorylated tau, Parkinson's disease

## Abstract

Deep brain stimulation (DBS) is an established and effective surgical treatment for movement disorders, including Parkinson's disease (PD). However, postmortem studies of patients with PD have revealed the presence of gliosis and inflammatory responses around the tracts of DBS electrodes. The objective of this study was to investigate the deposition of abnormal proteins, including phosphorylated tau (p-tau) and transactivation response DNA-binding protein 43 kDa (p-TDP-43), around the tips of DBS electrodes. Neuropathological examination was performed in two Japanese patients with PD: Case 1 describes a patient who underwent DBS lead placement into the left ventral intermediate nucleus of the thalamus at 79 years of age, and died at 88 years of age; Case 2 describes a patient who underwent DBS lead placement into the bilateral subthalamic nuclei at 70 years of age, and died 14 years after surgery. Postmortem neuropathological examination revealed fibrous gliosis, mild infiltration of lymphocytes, and hemosiderin deposition around the DBS lead tip-associated defects. Moreover, p-TDP-43 and p-tau deposits were visible around the electrode termination sites in both cases. These findings suggest that p-TDP-43 and p-tau accumulated around the DBS lead tip in response to chronic DBS. This is the first study to report the deposition of p-TDP-43 around the tip of a DBS electrode.

## Highlights

• Postmortem neuropathological examination was performed in two patients with Parkinson's disease.
• Both underwent implantation of deep brain simulation (DBS) leads.
• The deposition of p-tau was visible around the tips of the DBS leads in both cases.
• Deposition of p-TDP-43 was also found in the DBS lead tip-associated defects in both cases.
• Both p-TDP-43 and p-tau accumulated around the DBS lead tip in response to chronic and repetitive DBS.

## 1 Introduction

Deep brain stimulation (DBS) is an established and effective surgical technique for the treatment of movement disorders such as Parkinson's disease (PD) essential tremor disabling dystonia and Gilles de la Tourette syndrome [1]. Previous postmortem neuropathological studies of patients with PD who have undergone DBS treatment have revealed the presence of astrogliosis and chronic inflammatory responses surrounding the DBS electrode tracts [2]. These results indicate that chronic and repetitive DBS can induce reactive pathological changes. In this studywe report two cases of phosphorylated transactivation response DNA-binding protein of 43 kDa (p-TDP-43) accumulation around the tip of DBS electrodes for the first time.

## 2 Case presentation

The detailed clinical and pathological information is described in the Supplementary Information.

### 2.1 Case 1

A 73-year-old Japanese man presented with tremors in his right hand particularly while writing; 2 years later he exhibited bilateral upper limb rigidity and bradykinesia and was diagnosed with PD. Despite the initiation of oral trihexyphenidyl hydrochloride gabapentin and zonisamide the tremors in the right hand and jaw continued to gradually worsen. At 79 years of age he underwent stereotaxic implantation of a DBS electrode injected into the left ventral intermediate (Vim) nucleus of the thalamus. Brain computed tomography (CT) revealed a DBS lead tip in the rim nucleus of the thalamus (Fig. S1). After initiating DBS the tremor in his right hand improved drastically. At 80 years of age he began to exhibit signs of dysphagia and experienced aspiration pneumonia several times. His motor symptoms gradually deteriorated although he continued oral levodopa/carbidopa entacapone zonisamide and istradefylline. At 88 years of age the patient died of respiratory failure with a clinical course of approximately 9 years and 3 months after the implantation of the DBS electrode (15 years after symptom onset).

The brain weighed 1210 g before fixation and gross examination revealed mild atrophy of the bilateral frontal lobes (Supplementary Fig. S2). A small hole was visible on the left frontal cortical surface corresponding to the DBS electrode entry point (Supplementary Fig. S2). Depigmentation of the substantia nigra and locus coeruleus was visible in the brainstem. Microscopic examination revealed moderate-to-severe neuronal loss with gliosis particularly in the substantia nigra locus coeruleus and dorsal motor nucleus of the vagus nerve. Immunohistochemical staining with anti-phosphorylated α-synuclein (p-α-syn) antibodies revealed many p-α-*syn*-positive neuronal inclusions and neurites in the substantia nigra locus coeruleus dorsal motor nucleus of the vagus nerve and nucleus basalis of Meynert (NBM).

**Fig. 1:**
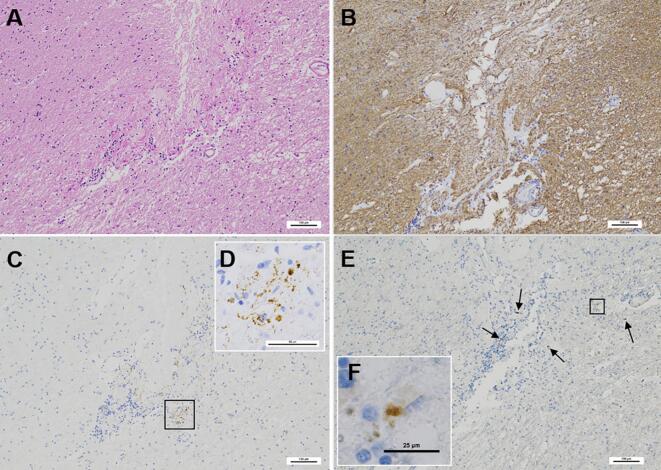
Microscopic findings in the deep brain stimulation electrode termination site (Case 1). (**A**) Prominent fibrous gliosis with Rosenthal fibers and mild infiltration of lymphocytes are visible around the cavity associated with the DBS lead tip in the Vim nucleus of the thalamus (HE staining). (**B**) Immunostaining with anti-GFAP antibodies shows astrogliosis around the tip of DBS electrode. (**C**–**D**) A small amount of p-tau-positive structures are visible in the immediate vicinity of the DBS lead tip (immunohistochemical staining for p-tau). (**D**) A high magnification of a square in (**C**). (**E–F**) Immunohistochemistry for p-TDP-43 is visualized with DAB. p-TDP-43-positive aggregates are visible near the tip of the DBS electrode (small arrows in **E**). (**F**) High magnifications of squares in (**E**). Scale bars = 100 μm in (**A**–**C–E**) 50 μm in (**D**) and 25 μm in (**F**).

In the Vim nucleus of the left thalamus prominent fibrous gliosis with Rosenthal fibers mild lymphocyte infiltration and mild hemosiderin deposition were observed around the DBS lead-tip-associated cavity (Fig. 1A). Immunostaining with an anti-glial fibrillary acidic protein (GFAP) antibody revealed increased astrogliosis surrounding the DBS track (Fig. 1B). Moreover p-tau- (Figs. 1C and D) and p-TDP-43- (Figs. 1E and F) immunopositive deposits were visible in the neuropil adjacent to the electrode termination site. No p-α-syn positive structures were observed at this site. No p-TDP-43-positive neuronal cytoplasmic inclusions glial cytoplasmic inclusions or dystrophic neurites (DNs) were visible in other brain regions and spinal cord. Moreover no TDP-43 depositions were observed in the right thalamus which was contralateral to the DBS lead-inserted side.

### 2.2 Case 2

A 51-year-old Japanese woman initially presented with gait disturbance. The patient was diagnosed with PD and the administration of L-dopa cabergoline and amantadine improved her motor symptoms. At 64 years of age the patient began to exhibit the wearing-off phenomenon and needed help when walking in the OFF state. At 70 years of age the patient underwent stereotaxic implantation of a DBS electrode in the bilateral subthalamic nuclei. Brain CT revealed the DBS lead tips in the bilateral subthalamic nuclei (Supplementary Fig. S1). After initiating DBS her motor symptoms especially akinesia improved. At 74 years of age she began to fall more frequently. Two years later her motor symptoms progressed to wheelchair-bound unless aided. At 84 years of age she died of multiple organ failure with a clinical course of approximately 14 years after implantation of the DBS electrodes (33 years from symptom onset).

The brain weighed 1122 g before fixation. A small hole was visible on the left frontal cortical surface corresponding to the DBS electrode entry point (Supplementary Fig. S2). Severe depigmentation of the substantia nigra and locus coeruleus was also observed. Microscopic examination revealed severe neuronal loss with gliosis and many p-α-*syn*-positive neuronal inclusions and neurites were visible in the substantia nigra locus coeruleus and dorsal motor nucleus of the vagus nerve. Lewy pathology was also observed in the olfactory bulb NBM amygdala transentorhinal cortex hippocampus and anterior cingulate gyrus; and the frontal temporal and occipital cortices albeit less frequently.

**Fig. 2:**
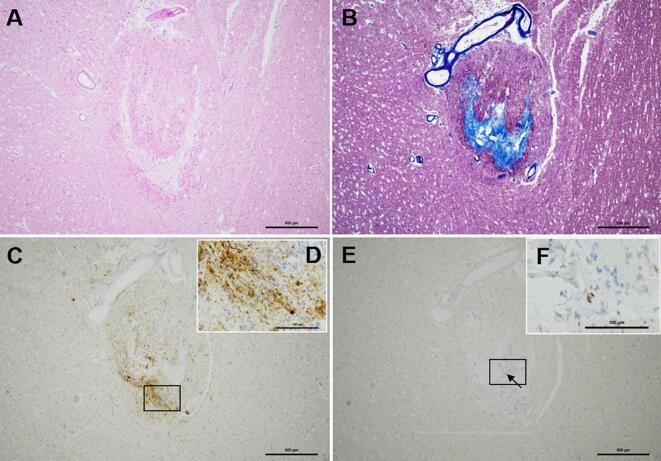
Microscopic findings in the deep brain stimulation electrode termination site (Case 2). (**A**–**B**) An increase of fibrous connective tissues with gliosis is visible around the cavity associated with the DBS lead tip in the left subthalamic nucleus via HE (**A**) and Azan staining (**B**). (**C**–**D**) A relatively high amount of p-tau-positive structures are visible in the immediate vicinity of the DBS lead tip (immunohistochemical staining for p-tau). (**D**) A high magnification of a square in (**C**). (**E–F**) Only tiny p-TDP-43-positive aggregates are visible near the tip of the DBS electrode (small arrow in **E**; immunohistochemical staining for p-TDP-43). (**F**) High magnifications of squares in (**E**). Scale bars = 500 μm in (**A–C–E**) 100 μm in (**D** and **F**).

In the left subthalamic nucleus prominent fibrous gliosis with mild infiltration of lymphocytes was visible around the DBS lead-tip-associated cavity (Figs. 2A and B). Immunostaining with an anti-GFAP antibody revealed increased astrogliosis surrounding the DBS track. Positive p-tau deposits were visible in the neuropil adjacent to the electrode termination site (Figs. 2C and D) when compared with those in Case 1 (Figs. 1C and D). Tiny p-TDP-43 positive structures were also observed at this site (Figs. 2E and F); however no p-TDP-43-positive structures were visible in other brain regions.

## 3 Discussion

In the present study p-TDP-43 and p-tau deposition were observed around the tip of the DBS electrodes. Notably no p-TDP-43-positive structures were observed in other regions of the brain including the spinal cord. Accumulation of p-TDP-43 and p-tau has been associated with chronic head injury [3] or massive cerebrovascular events [4]; abnormal aggregation of these proteins may therefore be associated with chronic external stimulation. This study sheds light on a novel mechanism of protein aggregation in the central nervous system.

Accumulation of p-TDP-43 in the central nervous system is found in neurodegenerative diseases such as amyotrophic lateral sclerosis (ALS) and frontotemporal lobar degeneration as well as in normal aging [5]. Uchino et al. examined TDP-43 pathology in 286 consecutive autopsy brains and found that p-TDP-43-positive DNs were predominantly observed in the uncus of the anterior hippocampus in control brains of the elderly [5]. Additionally p-TDP-43 positive structures were predominantly visible in the uncus of the anterior hippocampus and amygdala in patients with Lewy body disease [5]. Conversely Nelson et al. proposed a new neuropathological entity; i.e. limbic-predominant age-related TDP-43 encephalopathy (LATE) neuropathological change (NC) in 2019 [6]. They reported LATE-NC in 20–50 % of octogenarian patients and the LATE-NC staging system demonstrated that accumulation of p-TDP-43 initiates in the amygdala and extends to the hippocampus and middle frontal gyrus [6]. In ALS p-TDP-43 pathology initiates in the motor neocortex bulbar somatomotor neurons of nerve XII or ventral horn α-motoneurons in the spinal cord [7]. However no p-TDP-43-positive structures were observed in any brain regions in our study except for the Vim nucleus and subthalamic nucleus where the tip of DBS leads were inserted. These results suggest that the accumulation of p-TDP-43 around the DBS lead tip cannot be attributed to aging or other neurodegenerative processes.

Previous reports have shown that pathological changes around the tracks of DBS electrodes — including astrogliosis/elevated GFAP staining inflammatory changes such as the infiltration of T lymphocytes and/or activated microglia/macrophages iron deposition and axonal damage — are visible along the DBS electrode tracts [2]. Thus DBS can cause focal tissue damage. However few reports have indicated the deposition of p-tau near the DBS electrode tracts [8]. In our cases a small amount of p-tau was visible around the tip of the DBS electrode. Chronic traumatic encephalopathy is known to be associated with both neuronal and glial tau pathology [3] and in vivo studies have revealed that traumatic brain injury can induce the formation and spread of pathological tau proteins [9]. A previous study also reported a higher frequency of p-tau accumulation in the ipsilateral NBM in patients with an middle cerebral artery territory infarct or putaminal hemorrhage than of p-tau accumulation in the NBM-unaffected side [4]. These results suggest that cellular stress induced by cerebrovascular events including inflammation and oxidative stress may be responsible for the accumulation of p-tau. Moreover the deposition of p-TDP-43 was visible around the electrode termination sites in our study. The accumulation of both p-TDP-43 and p-tau has been noted in several brain regions of patients with a history of repetitive traumatic brain injury [3] as well as in those with massive cerebrovascular diseases [4]. In the present case report p-TDP-43-positive aggregates were visible in the neuropil and appeared as round-shaped neurites. Chronic repetitive and focal stimulation by DBS electrodes may be associated with prolonged cellular stress which can induce the abnormal aggregation of TDP-43 [10]. However the results revealed that p-tau and p-TDP-43 did not colocalize around the DBS lead tip suggesting that the mechanisms of deposition might differ between the two proteins.

In conclusion our results highlight a novel pathological change associated with DBS. Chronic and repetitive electric stimulation can cause the aggregation and deposition of abnormal proteins including p-TDP-43 and p-tau which may modify the physiological effects of DBS. Further cases of DBS should be examined to elucidate the underlying mechanisms.

1499 words.

## Approval of the research protocol

This study was approved by the Ethics Committee of Osaka University Hospital (No. 20043).

## Financial disclosure

The authors declare no Conflict of Interests for this article.

## CRediT authorship contribution statement

**Goichi Beck:** Writing – original draft, Visualization, Investigation, Funding acquisition, Conceptualization. **Yuki Yonenobu:** Visualization, Investigation. **Kenichiro Maeda:** Visualization, Investigation. **Satoru Oshino:** Investigation. **Haruhiko Kishima:** Investigation. **Masanori P. Takahashi:** Investigation. **Hideki Mochizuki:** Supervision, Investigation. **Shigeo Murayama:** Writing – review & editing, Investigation, Funding acquisition, Conceptualization.

## Informed consent

Written informed consent was obtained from the family of the patients described in this case report.

## Funding sources

This work was supported by the 10.13039/501100001691Japan Society for the Promotion of Science Grants in Aid for Scientific Research (KAKENHI) Grant Number JP20K06910 (to GB) Grant-in-Aid for Scientific Research on Innovative Areas Grant Number JP16H06277 (to SM) AMED under Grant Numbers JP21wm0425018 (to GB) JP20dm017106 (to SM) and JP21wm0425019 (to SM).

## Declaration of competing interest

None.

## Appendix A Supplementary data

Supplementary material Image 1

## References

[1] Krack P., Volkmann J., Tinkhauser G., Deuschl G. (2019). Deep brain stimulation in movement disorders: from experimental surgery to evidence-based therapy. Mov. Disord..

[2] Kronenbuerger M., Nolte K.W., Coenen V.A., Burgunder J.M., Krauss J.K., Weis J. (2015). Brain alterations with deep brain stimulation: new insight from a neuropathological case series. Mov. Disord..

[3] McKee A.C., Stern R.A., Nowinski C.J. (2013). The spectrum of disease in chronic traumatic encephalopathy. Brain.

[4] Hatsuta H., Takao M., Nogami A. (2019). Tau and TDP-43 accumulation of the basal nucleus of Meynert in individuals with cerebral lobar infarcts or hemorrhage. Acta Neuropathol. Commun..

[5] Uchino A., Takao M., Hatsuta H. (2015). Incidence and extent of TDP-43 accumulation in aging human brain. Acta Neuropathol. Commun..

[6] Nelson P.T., Dickson D.W., Trojanowski J.Q. (2019). Limbic-predominant age-related TDP-43 encephalopathy (LATE): consensus working group report. Brain.

[7] Brettschneider J., Del Tredici K., Toledo J.B. (2013). Stages of p-TDP-43 pathology in amyotrophic lateral sclerosis. Ann. Neurol..

[8] Okada K., Hata Y., Ichimata S. (2021). An autopsy case of pure nigropathy with TUBA4A nonsense mutation. Neuropathol. Appl. Neurobiol..

[9] Edwards G., Zhao J., Dash P.K., Soto C., Moreno-Gonzalez I. (2020). Traumatic brain injury induces tau aggregation and spreading. J. Neurotrauma.

[10] Ratti A., Gumina V., Lenzi P. (2020). Chronic stress induces formation of stress granules and pathological TDP-43 aggregates in human ALS fibroblasts and iPSC-motoneurons. Neurobiol. Dis..

