# Supplementary material for: Phosphorylated TDP-43 and tau deposition around the tip of deep brain stimulation leads
Source: eNeurologicalSci. 2025 May 13;39:100568. doi: 10.1016/j.ensci.2025.100568 (PMC12143835; doi:10.1016/j.ensci.2025.100568)
Supplement: Supplementary file 1 — Supplementary material [file mmc1.docx]

**Supplementary Information**

**Phosphorylated TDP-43 and tau deposition around the tip of deep brain stimulation leads**

Goichi Beck, Yuki Yonenobu, Kenichiro Maeda, Satoru Oshino, Haruhiko Kishima, Masanori P Takahashi, Hideki Mochizuki, Shigeo Murayama

***Correspondence**:

Goichi Beck M.D., Ph.D.

[g-beck@neurol.med.osaka-u.ac.jp](mailto:g-beck@neurol.med.osaka-u.ac.jp)

Shigeo Murayama M.D., Ph.D.

[smurayam@bbarjp.net](mailto:smurayam@bbarjp.net)

This file includes:

Clinical information

Materials & Methods

Pathological findings

Figures S1 & S2

Reference

**Clinical Information**

**Case #1**

A 73-year-old Japanese man presented with tremors in his right hand, particularly while writing; 2 years later, he exhibited bilateral upper limb rigidity and bradykinesia, and was diagnosed with PD. Brain MRI revealed no apparent atrophy of the putamen, brainstem, or cerebellum (Supplementary Figure S1A). After initiating levodopa/carbidopa treatment (300 mg/day), the tremors improved. At 76 years of age, the patient began to exhibit postural instability. Despite the initiation of oral trihexyphenidyl hydrochloride, gabapentin, and zonisamide, the tremors in the right hand and jaw continued to gradually worsen.

At the age of 79 years, the patient had no history of dementia, and his Mini-Mental State Examination (MMSE) score was 30. He underwent stereotaxic implantation of a DBS electrode (Model 3389; Medtronic, Minneapolis, MN, USA), injected into the left Vim nucleus of the thalamus. A model 7426 (Medtronic) was used as the implantable pulse generator. Brain CT revealed a DBS lead tip in the rim nucleus of the thalamus (Figure S1B). After initiating DBS (stimulation amplitude, 2.1–3.6 V; pulse width, 60–90 μs; frequency, 130–160 Hz), the tremor in his right hand improved drastically. At 80 years of age, he began to exhibit signs of dysphagia and experienced aspiration pneumonia several times. Two years later, his dysphagia and bradykinesia were significantly exaggerated, and nutritional support with percutaneous endoscopic gastrostomy was initiated. His motor symptoms gradually deteriorated, although he continued oral levodopa/carbidopa, entacapone, zonisamide, and istradefylline. However, the patient showed no cognitive impairment, and his HDS-R score was 29 at 87 years of age. Similar to the MMSE, the HDS-R is widely used to assess cognitive functions, especially in Japan and other Asian countries; a score <20 indicates the presence of dementia [1]. Moreover, no psychiatric symptoms, including hallucinations or delusions, were noted.

At 88 years of age, he was admitted to our hospital with aspiration pneumonia. Although antibiotic therapy was initiated, the patient died of respiratory failure with a clinical course of approximately 9 years and 3 months after the implantation of the DBS electrode (15 years after symptom onset). An autopsy was performed 3 h and 57 min after death.

**Case #2**

A 51-year-old Japanese woman initially presented with gait disturbance; she visited the neurology department 1 year later, and a neurological examination revealed rigidity and akinesia, predominantly in the right upper and lower limbs. The patient was diagnosed with PD, and the administration of L-dopa, cabergoline, and amantadine improved her motor symptoms. Brain MRI showed no abnormalities. At 64 years of age, the patient began to exhibit the wearing-off phenomenon and needed help when walking in the OFF state; thus, the L-dopa dose was increased to 600 mg/day. At 68 years of age, the patient started to show L-dopa-induced dyskinesia; the dose of L-dopa was therefore decreased, and a COMT inhibitor was initiated. A brain MRI revealed no apparent atrophy of the putamen, brainstem, or cerebellum (Supplementary Figure S1C).

At 70 years of age, the patient underwent stereotaxic implantation of a DBS electrode in the bilateral subthalamic nuclei. Brain CT revealed the DBS lead tips in the bilateral subthalamic nuclei (Supplementary Figure S1D). After initiating DBS (stimulation amplitude, 2.3–3.0 V; pulse width, 60–90 μs; frequency, 130 Hz), her motor symptoms, especially akinesia, improved; the MDS-UPDRS motor examination (part III) score in the ON state improved from 29 to 10.

At 74 years of age, she began to fall more frequently. Two years later, she could not stand up and walk without help, and was admitted to a nursing home at 76 years of age; her motor symptoms progressed to wheelchair-bound unless aided (Hoehn and Yahr stage =5). At 84 years of age, she developed a high fever and abdominal distension, and was admitted to the hospital. Abdominal CT and blood tests revealed a large hepatic cyst infection. Percutaneous transhepatic drainage and administration of antibiotics temporarily improved her symptoms; however, she died of multiple organ failure with a clinical course of approximately 14 years after implantation of the DBS electrodes (33 years from symptom onset). An autopsy was performed 17 h and 25 min after death.

**Materials & Methods**

The right hemisphere of the brain was frozen for molecular and biochemical studies, while the left hemisphere and spinal cord were fixed in 10% buffered formalin. The right thalamus, located contralateral to the side where the DBS lead was inserted, was also fixed in 10% buffered formalin in Case #1. After macroscopic observation, appropriate areas were dissected and embedded in paraffin. Serial sections (6 μm thickness) were stained with hematoxylin and eosin. For immunohistochemistry, the primary antibodies used were phosphorylated α-synuclein (p-α-syn) (mouse monoclonal, clone pSyn#64, Fujifilm-Wako Industrial; 1:20000), phosphorylated tau (p-tau) (mouse monoclonal, clone AT8 IGH135; Cosmo Bio Co.; 1:800), TDP-43 phosphorylated at serine 409/410 residues (p-TDP-43) (mouse monoclonal, clone 11-9, Cosmo Bio Co.; 1:40000), amyloid-β (mouse monoclonal, clone 12B2; IBL; 1:100), glial fibrillary acidic protein (GFAP) (mouse monoclonal, NCL-L-GFAP-GA5, Leica Biosystems; 1:1000), CD3 (mouse monoclonal, clone F7.2.38, Dako; 1:1000), CD68 (mouse monoclonal, clone PG-M1, Dako; 1:1000), and Iba-1 (rabbit polyclonal, 019-19741, Wako; 1:500). Hematoxylin was used as the counterstain.

**Pathological findings**

**Case #1**

The brain weighed 1,210 g before fixation, and gross examination revealed mild atrophy of the bilateral frontal lobes (Supplementary Figure S2A and B). A small hole was visible on the left frontal cortical surface, corresponding to the DBS electrode entry point (Supplementary Figure S2A and B). Depigmentation of the substantia nigra and locus coeruleus was visible in the brainstem. Microscopic examination revealed moderate-to-severe neuronal loss with gliosis, particularly in the substantia nigra, locus coeruleus, and dorsal motor nucleus of the vagus nerve. Immunohistochemical staining with anti-p-α-syn antibodies revealed many p-α-syn-positive neuronal inclusions and neurites in the substantia nigra, locus coeruleus, dorsal motor nucleus of the vagus nerve, and NBM. Lewy pathology was also observed in the olfactory bulb, amygdala, transentorhinal cortex, hippocampus, and anterior cingulate gyrus. The neuropathological diagnosis was PD, classified as stage 4 according to the Braak staging system [2], and “limbic” according to the third DLB consensus guideline [3]. In the peripheral nervous system, Lewy pathology occurred in the myenteric plexus of the stomach, cardiac sympathetic nerves, and paraadrenal sympathetic ganglia.

In the Vim nucleus of the left thalamus, prominent fibrous gliosis with Rosenthal fibers, mild lymphocyte infiltration, and mild hemosiderin deposition were observed around the DBS lead-tip-associated cavity. Immunostaining with an anti-GFAP antibody revealed increased astrogliosis surrounding the DBS track. Immunohistochemical analysis of CD3, CD68, and Iba-1 revealed the infiltration of T lymphocytes and activated microglia/macrophages around the electrode termination site. Moreover, p-tau- and p-TDP-43-immunopositive deposits were visible in the neuropil adjacent to the electrode termination site. No p-α-syn positive structures were observed at this site.

No p-TDP-43-positive neuronal cytoplasmic inclusions, glial cytoplasmic inclusions, or DNs were visible in other brain regions, including the upper/middle/lower frontal cortex, precentral gyrus (primary motor cortex), upper/middle/lower temporal gyrus, entorhinal cortex, anterior cingulate gyrus, rectal gyrus, amygdala, hippocampus, nucleus accumbens, NBM, caudate nucleus, putamen, globus pallidus, subthalamic nucleus, supramarginal gyrus, visual cortex, substantia nigra, red nucleus, locus coeruleus, pontine nucleus, reticular formation of the pons and medulla oblongata, hypoglossal nucleus, dorsal motor nucleus of the vagus, inferior olivary nucleus, pyramids of medulla oblongata, and dentate nucleus of the cerebellum. Similarly, no p-TDP-43-positive structures were visible in the anterior horn, posterior horn, central gray matter, or white matter of the cervical and lumbar spinal cords. The brain and spinal cord regions included all areas used for assessing TDP-43 pathology according to the Brettschneider staging system or LATE stage. Moreover, no TDP-43 depositions were observed in the right thalamus, which was contralateral to the DBS lead-inserted side.

Additionally, p-tau immunohistochemistry revealed neurofibrillary tangles and threads in the hippocampus, entorhinal cortex, and transentorhinal cortex, indicating Braak AT8 stage II. Immunohistochemistry analysis for Aβ revealed diffuse types of senile plaques in the hippocampus alone.

**Case #2**

The brain weighed 1,122 g before fixation. A small hole was visible on the left frontal cortical surface, corresponding to the DBS electrode entry point (Supplementary Figure S3C). Severe depigmentation of the substantia nigra and locus coeruleus was also observed. Microscopic examination revealed severe neuronal loss with gliosis, and many p-α-syn-positive neuronal inclusions and neurites were visible in the substantia nigra, locus coeruleus, and dorsal motor nucleus of the vagus nerve. Lewy pathology was also observed in the olfactory bulb, NBM, amygdala, transentorhinal cortex, hippocampus, and anterior cingulate gyrus; and the frontal, temporal, and occipital cortices, albeit less frequently. The neuropathological diagnosis was PD, classified as stage 6 according to the Braak staging system [2], and as “diffuse neocortical” according to the third DLB consensus guideline [3]. In the peripheral nervous system, Lewy cell pathology occurs in the myenteric plexus of the stomach, cardiac sympathetic nerves, and paraadrenal sympathetic ganglia.

In the left subthalamic nucleus, prominent fibrous gliosis with mild infiltration of lymphocytes was visible around the DBS lead-tip-associated cavity. Immunostaining with an anti-GFAP antibody revealed increased astrogliosis surrounding the DBS track. Positive p-tau deposits were visible in the neuropil adjacent to the electrode termination site when compared with those in Case 1. Tiny p-TDP-43 positive structures were also observed at this site; however, no p-TDP-43-positive neuronal cytoplasmic inclusions, glial cytoplasmic inclusions, or DNs were visible in other brain regions, including the upper/middle/lower frontal cortex, precentral gyrus, upper/middle/lower temporal gyrus, entorhinal cortex, anterior cingulate gyrus, rectal gyrus, amygdala, hippocampus, nucleus accumbens, NBM, caudate nucleus, putamen, globus pallidus, subthalamic nucleus, supramarginal gyrus, visual cortex, substantia nigra, red nucleus, locus coeruleus, pontine nucleus, reticular formation of the pons and medulla oblongata, hypoglossal nucleus, dorsal motor nucleus of the vagus, inferior olivary nucleus, pyramids of medulla oblongata, and dentate nucleus of the cerebellum. No p-TDP-43-positive structures were visible also in the anterior horn, posterior horn, central gray matter, or white matter of the cervical and lumbar spinal cords.

By contrast, p-tau immunohistochemistry revealed NFTs and NTs in the transentorhinal cortex and occipitotemporal gyrus, but not in the medial temporal gyrus, indicating Braak AT8 stage III. Aβ immunohistochemical analysis revealed that SPs were frequently observed in brain areas including the precentral gyrus, anterior cingulate gyrus, and substantia nigra (CERAD stage B, and Thal phase 4).


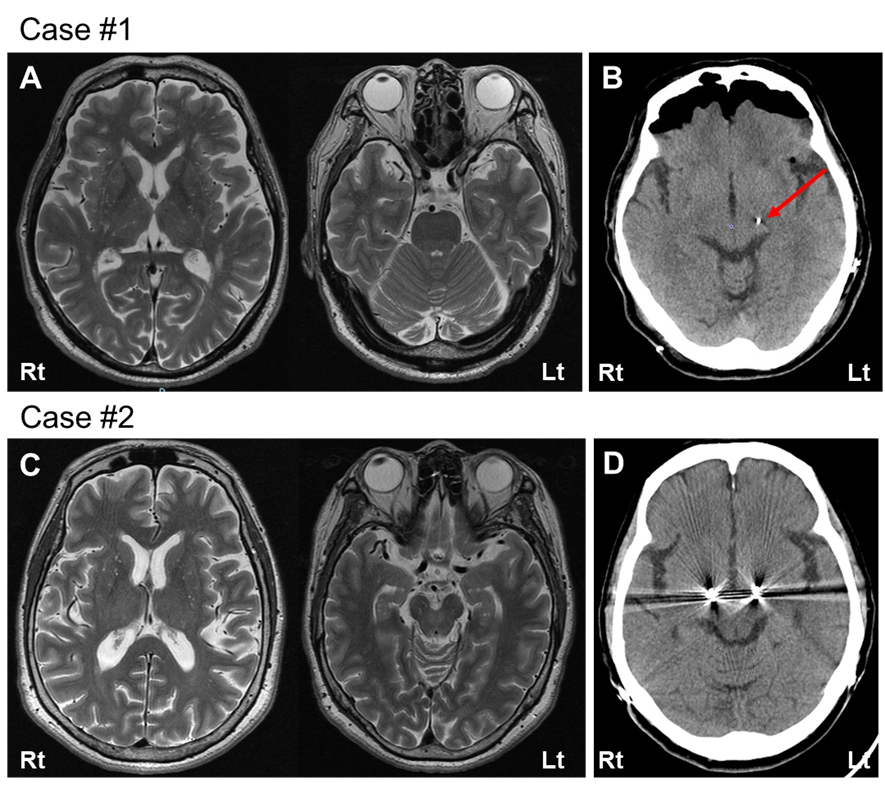


**Supplementary Figure S1. Radiological ﬁndings in the brain**

(A, B) Case 1

(A) Brain magnetic resonance T2-weighted images obtained at 75 years of age show no atrophy in the putamen, brainstem, or cerebellum.

(B) Brain CT after implantation of the deep brain stimulation electrode shows the presence of the tip of the electrode in the Vim nucleus of the left thalamus (red arrow).

(C, D) Case 2

(C) Brain magnetic resonance T2-weighted images performed at 68 years of age reveal no apparent atrophy in the putamen, brainstem, or cerebellum.

(D) Brain CT after implantation of the deep brain stimulation electrodes shows the presence of the tips of the electrodes in the bilateral subthalamic nuclei.


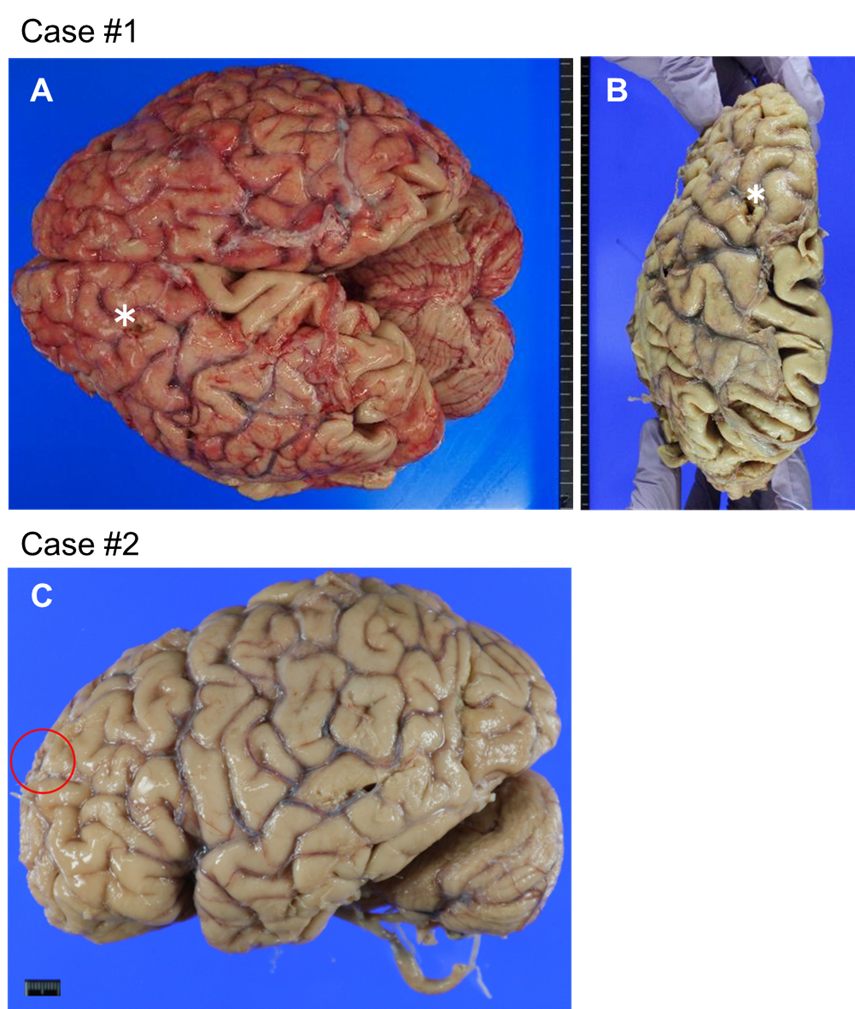


**Supplementary Figure S2. Macroscopic findings in the brain**

(A, B) Case 1

Convex external view of the cerebrum showing mild atrophic changes in the bilateral frontal lobe. A small hole is visible on the left frontal cortical surface corresponding to the entry point of the deep brain stimulation electrode (*).

(C) Case 2

A small hole is visible on the left frontal cortical surface, corresponding to the DBS electrode entry point (red circle).

**Reference**

1. Imai Y, Hasegawa K. The revised Hasegawa’s Dementia Scale (HDS-R) - evaluation of its usefulness as a screening test for dementia. J Hong Kong Coll Psychiatr 1994;4:20-24.
2. Braak H, Del Tredici K, Rüb U, de Vos RA, Jansen Steur EN, Braak E. Staging of brain pathology related to sporadic Parkinson's disease. Neurobiol Aging 2003;24:197-211.
3. McKeith IG, Dickson DW, Lowe J, Emre M, O'Brien JT, Feldman H, Cummings J, Duda JE, Lippa C, Perry EK, Aarsland D, Arai H, Ballard CG, Boeve B, Burn DJ, Costa D, Del Ser T, Dubois B, Galasko D, Gauthier S, Goetz CG, Gomez-Tortosa E, Halliday G, Hansen LA, Hardy J, Iwatsubo T, Kalaria RN, Kaufer D, Kenny RA, Korczyn A, Kosaka K, Lee VM, Lees A, Litvan I, Londos E, Lopez OL, Minoshima S, Mizuno Y, Molina JA, Mukaetova-Ladinska EB, Pasquier F, Perry RH, Schulz JB, Trojanowski JQ, Yamada M; Consortium on DLB. Diagnosis and management of dementia with Lewy bodies: third report of the DLB Consortium. Neurology 2005;65:1863-1872.
